# Regulatory Protein OmpR Influences the Serum Resistance of *Yersinia enterocolitica* O:9 by Modifying the Structure of the Outer Membrane

**DOI:** 10.1371/journal.pone.0079525

**Published:** 2013-11-19

**Authors:** Karolina Skorek, Adrianna Raczkowska, Bartłomiej Dudek, Katarzyna Miętka, Katarzyna Guz-Regner, Aleksandra Pawlak, Elżbieta Klausa, Gabriela Bugla-Płoskońska, Katarzyna Brzostek

**Affiliations:** 1 Department of Applied Microbiology, Faculty of Biology, University of Warsaw, Warsaw, Poland; 2 Department of Microbiology, Faculty of Biological Sciences, University of Wroclaw, Wroclaw, Poland; 3 Regional Centre of Transfusion Medicine and Blood Bank, Wroclaw, Poland; Arizona State University, United States of America

## Abstract

The EnvZ/OmpR two-component system constitutes a regulatory pathway involved in bacterial adaptive responses to environmental cues. Our previous findings indicated that the OmpR regulator in *Yersinia enterocolitica* O:9 positively regulates the expression of FlhDC, the master flagellar activator, which influences adhesion/invasion properties and biofilm formation. Here we show that a strain lacking OmpR grown at 37°C exhibits extremely high resistance to the bactericidal activity of normal human serum (NHS) compared with the wild-type strain. Analysis of OMP expression in the *ompR* mutant revealed that OmpR reciprocally regulates Ail and OmpX, two homologous OMPs of *Y. enterocolitica*, without causing significant changes in the level of YadA, the major serum resistance factor. Analysis of mutants in individual genes belonging to the OmpR regulon (*ail*, *ompX*, *ompC* and *flhDC*) and strains lacking plasmid pYV, expressing YadA, demonstrated the contribution of the respective proteins to serum resistance. We show that Ail and OmpC act in an opposite way to the OmpX protein to confer serum resistance to the wild-type strain, but are not responsible for the high resistance of the *ompR* mutant. The serum resistance phenotype of *ompR* seems to be multifactorial and mainly attributable to alterations that potentiate the function of YadA. Our results indicate that a decreased level of FlhDC in the *ompR* mutant cells is partly responsible for the serum resistance and this effect can be suppressed by overexpression of *flhDC* in *trans*. The observation that the loss of FlhDC enhances the survival of wild-type cells in NHS supports the involvement of FlhDC regulator in this phenotype. In addition, the *ompR* mutant exhibited a lower level of LPS, but this was not correlated with changes in the level of FlhDC. We propose that OmpR might alter the susceptibility of *Y. enterocolitica* O:9 to complement-mediated killing through remodeling of the outer membrane.

## Introduction

The human enteropathogen *Yersinia enterocolitica* is the causative agent of yersiniosis: an acute or chronic zoonotic disease producing a variety of clinical symptoms, i.e. intestinal, pseudo-appendicular, erythematous and septicaemic [1]. After *Campylobacter jejuni* and *Salmonella* spp., *Y. enterocolitica* is the third most common enteric pathogen associated with foodborne infections in Europe [2]. Before *Y. enterocolitica* infects a mammalian host the bacteria must survive free-living in the environment. After ingestion, *Yersiniae* cells have to adapt to the host body temperature (37°C), and survive in the face of various unfavorable environmental factors and the immune system. Switching between distinct niches within and outside the host presents *Y. enterocolitica* with a constant adaptive challenge. In response to different environmental cues, the pathogenic *Y. enterocolitica* synthesizes several chromosomal- and plasmid (pYV)-encoded virulence factors whose expression is tightly regulated [3], [4].

The molecular mechanisms of bacteria mediating adaptive changes in response to environmental signals are centered on two-component regulatory systems (TCSs) [5]. TCSs allow bacteria to modulate the expression of certain genes in response to fluctuations in various environmental cues to alter their physiology [6] and pathogenicity [7]. TCSs are widespread in bacteria including *Y. enterocolitica*. Putative TCSs of *Y. enterocolitica* have been identified based on theoretical relationships, although the function of most of them has yet to be defined [8].

Much of our knowledge of TCS regulation has come from studies on the prototypical two-component system EnvZ/OmpR of non-pathogenic *Escherichia coli* K-12, where EnvZ is the sensor kinase and OmpR the response regulator functioning as a transcription factor. Both proteins are involved in the reciprocal regulation of the *ompF* and *ompC* porin genes in response to changes in the osmolarity of the environment [9]. Besides porin genes, the *E. coli* OmpR response regulator has been shown to regulate other targets [10]. The EnvZ/OmpR system of *Y. enterocolitica* was initially identified due to its role in the regulation of porin expression. Homologs of the OmpC and OmpF porins were identified in the outer membrane of *Y. enterocolitica* and the pore forming activity of the OmpC was demonstrated in black lipid bilayer experiments [11], [12]. Moreover, the role of both porins in the permeability of the outer membrane and resistance of *Yersinia* cells to β-lactam antibiotics was confirmed [13], [14], [15]. Further studies revealed the relationship between virulence and the activity of the OmpR protein in *Y. enterocolitica* serotypes O:8 and O:9 [15], [16]. More detailed studies identified a role for OmpR in the inverse regulation of invasin and flagella expression in *Y. enterocolitica* O:9 [17], [18] and confirmed the involvement of OmpR in biofilm formation and the adherent-invasive abilities of this bacterium [19], [20]. In the present study we examined the role of the OmpR regulator in the ability of *Y. enterocolitica* O:9 to resist serum-dependent killing.

Serum resistance has been shown to be critical for the survival of invading bacteria and the establishment of disease [21]. A relevant phenotype for *Y. enterocolitica* survival in the host body, namely the ability to withstand the activity of complement-dependent killing, has been described in different bio-serotypes. The two groups of virulent serotypes, the so-called American (O:8, O:4,32, O:20) and non-American (O:3, O:9, O:5,27) groups, differ in their geographic distribution, host range, biochemical abilities and clinical manifestation [22]. Extensive studies, mostly performed on serotypes O:8 and O:3, led to the conclusion that YadA and Ail, two outer membrane proteins of *Y. enterocolitica*, are the factors primarily involved in the serum-resistant phenotype [23]. In addition, lipopolysaccharide (LPS), a major component of the outer leaflet of the outer membrane, may be indirectly involved in this phenomenon [24], [25].

The objective of this study was to characterize the role of *Y. enterocolitica* OmpR in mitigating the bactericidal activity of serum by examining strains differing in their content of OmpR and outer membrane proteins, and correlating the findings with variations in their serum susceptibility. Based on the association between the sensitivity to NHS of defined mutant strains and the OmpR-related phenotype, we determined that the OmpR regulator is responsible for controlling the susceptibility of *Y. enterocolitica* to complement-dependent killing. Our efforts to understand the role of OmpR in the serum resistance of *Y. enterocolitica* Ye9 showed for the first time that this regulator can influence expression of the homologous genes encoding outer membrane proteins Ail and OmpX, the former of which confers serum resistance. The results also revealed that the OmpC porin, encoded by another gene belonging to the OmpR regulon, affects serum susceptibility of *Y. enterocolitica* O:9. Despite these interesting findings, the results argued against roles for Ail and OmpC in the serum resistance of the *ompR* mutant AR4. Instead, our data suggested that the *ompR* mutation might potentiate the function of YadA, the major serum resistance factor, by affecting the LPS content and the expression of FlhDC, the regulator of the membrane-anchored flagellar type III secretion apparatus. This remodeling of the OM could alter the susceptibility of *Y. enterocolitica* O:9 to complement-mediated killing.

## Results

### The *ompR* null mutation influences the susceptibility of *Y. enterocolitica* Ye9 to complement-dependent killing

**Figure 1:**
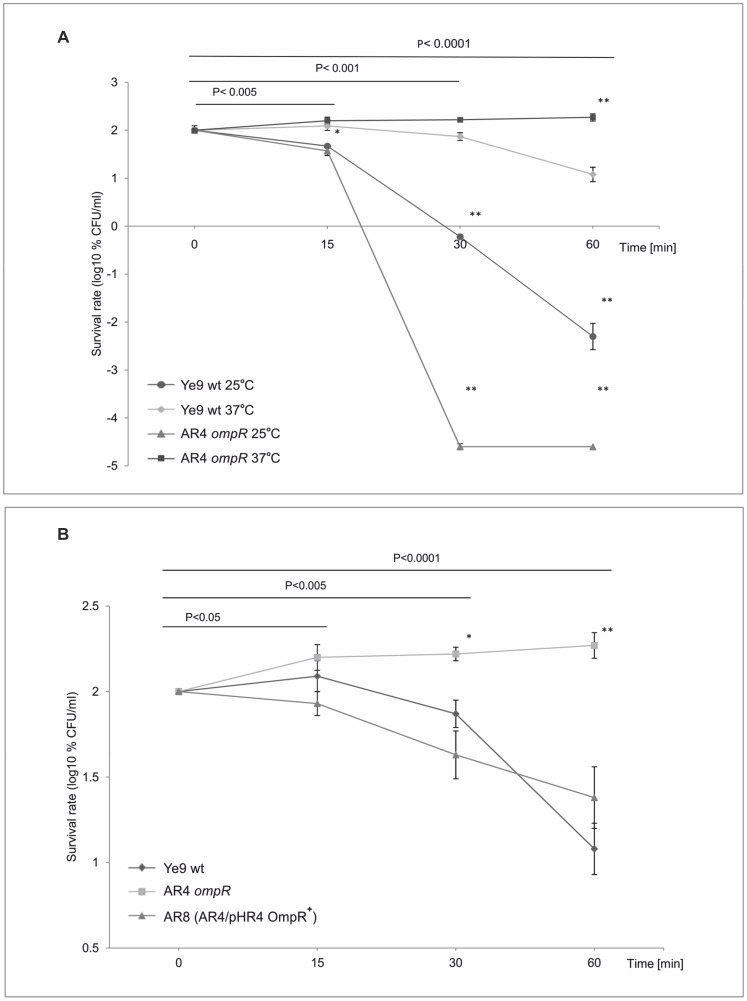
The susceptibility of *Y. enterocolitica* O:9 strains to normal human serum. Strains were grown in LB medium to exponential phase (OD_600_ of 0.3) and then incubated with 50% NHS at 37°C. Aliquots removed at 0, 15, 30 and 60 min were plated for viability counts (CFU). The numbers of CFU/ml were normalized to the T_0_ values as a percentage and then converted to log_10_ to show the survival rate of the bacteria in NHS. Data are the means ± SD from three independent experiments. **A**. The survival rate of Ye9 (wt) and AR4 (*ompR*) strains in NHS. Strains were grown at 25 or 37°C. The asterisks indicate statistically significant differences between strains and temperature conditions (* P<0.005, ** P<0.0001, by two-way repeated measures ANOVA with Tukey's comparision post-test). **B**. The survival rate of *Y. enterocolitica* O:9 strains with and without OmpR in NHS. Strains Ye9 (wt), AR4 (*ompR*) and AR8 (*ompR* mutant AR4 with plasmid pHR4 OmpR^+^) were grown at 37°C. The asterisks indicate statistically significant differences between strains (* P<0.005, ** P<0.0001, by one-way repeated measures ANOVA with Tukey's comparison post-test).

Using an *ompR* mutant (Δ*ompR*::Km) of *Y. enterocolitica* Ye9 constructed by a reverse genetics PCR-based strategy, we previously showed that OmpR is involved in the response of this bacterium to environmental stresses, and reciprocally regulates invasion and motility, which may confer an advantage in particular ecological niches [15], [17], [18], [19]. Another important property of enteropathogens is their ability to resist the bactericidal effects of normal human serum (NHS) and experiments were performed to determine whether OmpR can also influence the susceptibility of *Y. enterocolitica* Ye9 to complement-dependent killing. First, the serum sensitivity of the wild-type (wt) strain Ye9, grown either at 25 or 37°C in LB medium, was determined by incubation at 37°C with 50% NHS for 0, 15, 30 and 60 min (Fig. **1A**). Ye9 cells grown at 25°C were highly susceptible to NHS. In contrast, Ye9 grown at 37°C displayed resistance to killing by 50% NHS, and after 15-min incubation this strain exhibited ∼124% survival. However, analysis of the killing rate demonstrated a stepwise reduction in the viability of strain Ye9, and after 60-min incubation with NHS, survival had decreased to ∼12% (P<0.0001) (Fig. **1A**). This serum resistance phenotype of strain Ye9 was described as intermediate/moderate (Table S1). The same level of serum resistance was observed with the Nal^R^ derivative of Ye9 (data not shown). The bactericidal activity of NHS was not detected if the serum was heat-inactivated prior to the assay (HIS), indicating that the mechanism of killing Ye9 cells was complement-mediated (Table S2). In all cases, cells of the examined strains multiplied, and treatment with HIS was used as a bacterial multiplication control.

Strain AR4, a null mutant of *ompR* derived from Ye9, was then examined for survival in NHS. When grown at 25°C, the viability of AR4 decreased dramatically upon incubation with 50% NHS (Fig. **1A**). While after 15 min of treatment, the survival of AR4 was comparable with that of Ye9, a far higher degree of killing was observed for AR4 compared to the wild type after incubation for 60 min (P<0.0001). In contrast, the assay performed with strain AR4 grown at 37°C revealed that the *ompR* null mutation increased survival in 50% NHS. After 60-min incubation, survival increased to reach 186% and was ∼15-fold higher than that of the Ye9 wild type. The serum survival assay was also performed with mutant strain AR4 carrying the gene coding for OmpR on plasmid pHR4, grown at 37°C (Fig. **1B**). Complementation of the *ompR* mutation (strain AR8) resulted in a significant decrease in survival after 60-min incubation with NHS (25%). These results revealed that OmpR is involved in complement-mediated serum killing. In addition, the experiments confirmed the temperature-dependent serum resistance phenotype of *Y. enterocolitica*, although the primary thermoregulatory phenomenon seems to be OmpR-independent.

To pinpoint the nature of the OmpR-regulatory mechanism, several *Y. enterocolitica* strains differing in their outer membrane protein content (derivatives of strains Ye9 and AR4) were constructed, grown at 37°C and tested for their susceptibility to complement-dependent killing by incubation with NHS and HIS (as a control) at 37°C. These strains exhibited varying degrees of serum resistance summarized in Tables S1 and S2. Below, we present the results of experiments aimed at explaining the serum resistance phenotype of *ompR* mutant strain AR4 grown at 37°C.

### Analysis of OmpR-dependent outer membrane protein content

**Figure 2:**
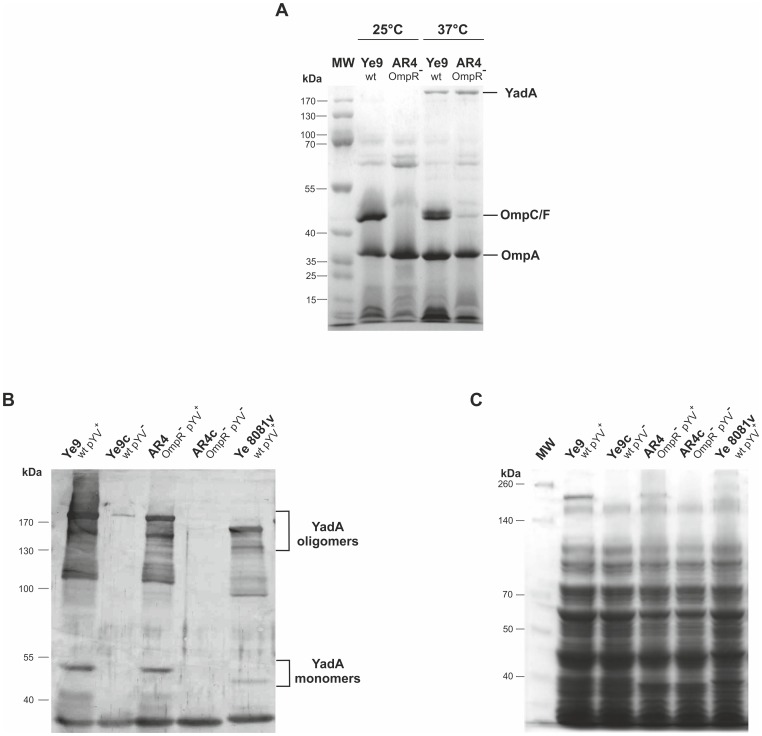
Analysis of outer membrane proteins of *Y. enterocolitica* O:9. **A**. Outer membrane fractions were isolated from bacterial cells grown in LB broth to an OD_600_ of 1.0 at either 25 or 37°C. Coomassie blue-stained SDS-polyacrylamide gel showing OMPs isolated from strains Ye9 and AR4. The positions of the OmpC/F porins, OmpA and YadA are indicated. MW, molecular weight standards (PageRuler Prestained Protein Ladder). The gel shown is representative of the results of an experiment performed several times. **B**. Whole-cell lysates of different strains, with or without plasmid pYV (pYV^+^/^−^), grown at 37°C to exponential phase were separated by SDS-PAGE and immunoblot analysis was performed using polyclonal YadA antiserum. YadA protein oligomers and monomers are indicated. This experiment was performed three times with similar results. **C**. Coomassie blue-stained SDS-polyacrylamide gel of whole-cell lysates of strains used for immunoblot analysis to show loading. MW, molecular weight standards (PageRuler Prestained Protein Ladder).

The serum survival assays suggested that a lack of OmpR may affect factors localized in the bacterial outer membrane (OM). Therefore, we analyzed the outer membrane protein (OMP) profiles of different *Y. enterocolitica* strains. The outer membrane protein YadA (yersinia adhesion A), in its homotrimeric structure of ∼200 kDa, performs multiple functions in *Yersinia*, including a crucial role in protecting enteropathogenic strains of *Y. enterocolitica* (grown at 37°C) against complement-mediated killing [24], [26], [27]. This protein is encoded by the virulence plasmid pYV and expressed at very high levels at 37°C [28]. The presence of YadA in the OM of strains Ye9 and AR4 carrying pYV and in isogenic derivatives lacking pYV (Ye9c and ARc) was studied by SDS-PAGE and immunoblot analysis (Fig. **2A, B, C**). The lack of plasmid pYV in the cured strains was confirmed by PCR with primers yH1 and yH517 that generate a 517-bp amplicon (data not shown). Some difficulties were encountered in obtaining plasmid-cured cells of strain AR4 (AR4c). Moreover, when grown in BHI broth, AR4c exhibited an altered cellular morphology compared to its plasmid-bearing parent. At 37°C, plasmid-containing strain AR4 grew as rods in short chains, whereas the cured strain AR4c appeared as single short rods (data not shown). Analysis of the outer membrane protein profiles of *Y. enterocolitica* strains Ye9 and AR4 (both carrying pYV) demonstrated that temperature and the presence of the OmpR regulator had a visible impact on the OMP content (Fig. **2A**). The OmpC/OmpF porins (a single band in cells grown at 25°C and two bands in cells grown at 37°C) and the OmpA protein were found predominately in the OM fraction of Ye9 at both temperatures. However, the OmpF/OmpC porins were absent from the OM of *ompR* strain AR4 grown at 25°C and 37°C (a faint unidentified protein band at the OmpF/OmpC position was sometimes detected in cells of this strain grown at 37°C). The level of OmpA in the OM fraction of strain AR4 was not affected by the growth temperature and was comparable with that detected in Ye9 OM samples. These data corroborated the results of a previous study which suggested that OmpC/F porin expression is OmpR-dependent [17]. The OMP profiles of strains Ye9 and AR4 grown at 37°C also contained similar levels of the high molecular weight pYV-encoded YadA outer membrane protein migrating at approximately 200 kDa, which was not visible in outer membrane samples of the same strains grown at 25°C (Fig. **2A**). The presence of *Y. enterocolitica* YadA in these samples was confirmed by excising the selected protein band from the gel and subjecting it to liquid chromatography-tandem mass spectrometry analysis (LC MS/MS, data not shown). Levels of YadA in whole-cell extracts of strains Ye9 and AR4, with and without pYV, were analyzed by immunoblotting using polyclonal YadA antiserum. As shown in Figure **2B**, all strains carrying pYV displayed a monomeric form of YadA migrating at approximately 50 kDa and a smear of oligomeric forms extending up to ∼200 kDa. Based on the signal intensities, there was no significant difference in the level of YadA between the analyzed strains. To compare the YadA level in different *Y. enterocolitica* serotypes (O:9 and O:8), strain Ye 8081v (serotype O:8) was included in the analysis. The size of YadA monomers in Ye 8081v appeared to be slightly smaller than the Ye9 YadA monomer, confirming the known YadA polymorphism between strains/serotypes of this bacterium [27], [29].

### Serum-sensitive phenotype of *Y. enterocolitica* O:9 strains lacking protein YadA

**Figure 3:**
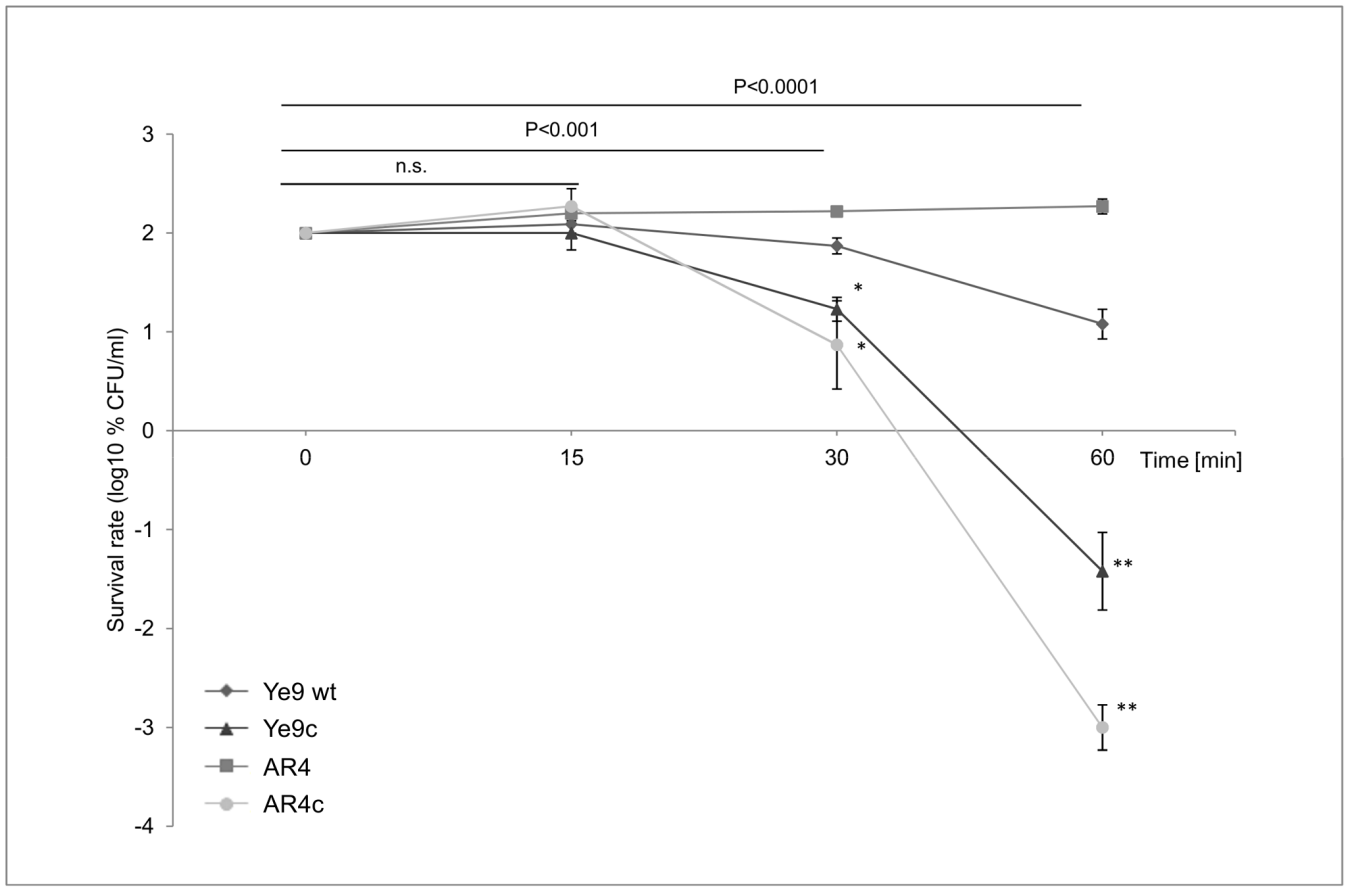
The susceptibility of *Y. enterocolitica* O:9 strains lacking YadA protein to normal human serum. Strains Ye9 (wt) and AR4 (*ompR*) harboring the virulence plasmid pYV (*yadA* ^+^) and their plasmid-cured derivatives (Ye9c and AR4c, respectively) were grown to exponential phase at 37°C and incubated with 50% NHS at 37°C. Aliquots removed at 0, 15, 30 and 60 min were plated for viability counts (CFU). The numbers of CFU/ml were normalized to the T_0_ values as a percentage and then converted to log_10_ to show the survival rate of the bacteria in NHS. Data are the means ±SD from at least three independent experiments. The Ye9c, pYV^−^ and the AR4c, pYV^−^ asterisks indicate statistically significant difference in comparison with the strains carrying pYV (Ye9 and AR4) (* P<0.001, ** P<0.0001, by one way ANOVA with Tukey's comparison post-test), n.s. not significant.

To assess the role of OmpR in YadA-mediated serum resistance of *Y. enterocolitica* Ye9, serum susceptibility assays were performed with pairs of strains differing in their plasmid content (pYV^+^ and pYV^−^), grown at 37°C. The data presented in Fig. **3** revealed pronounced differences between these strains. While Ye9 wt exhibited moderate resistance to the bactericidal action of human serum (12% survival after 60-min incubation), strain Ye9c without plasmid pYV-encoded YadA was highly susceptible to complement-mediated killing (∼300-fold lower serum resistance at 60 min) compared to the parent strain (P<0.0001). Strain AR4 lacking OmpR (YadA-positive) displayed high resistance in 50% NHS, while the plasmid pYV-cured derivative AR4c (YadA-negative) showed a dramatic (∼10,000-fold) decrease in serum resistance, particularly after 60-min incubation with NHS (P<0.0001). The survival percentage of AR4c fell significantly below that of Ye9c (P<0.0001) (Fig. **3**). In the light of these results and previous data it is apparent that the primary temperature-dependent change in serum resistance is YadA-dependent. These experiments also provided evidence that YadA is important for the serum resistance of both the Ye9 and AR4 strains of *Y. enterocolitica*. However, the loss of pYV, expressing YadA, by plasmid curing, resulted in a greater (33-fold) decrease in serum resistance in the *ompR* mutant AR4 than in the wild-type Ye9. Since SDS-PAGE analysis showed no significant differences in the level of YadA in the OM of AR4 and Ye9, it seems that the *ompR* mutation in the former strain could potentiate YadA function.

### OmpR-dependent *ail* expression in *Y. enterocolitica* Ye9

Previous studies have demonstrated that besides the major serum resistance factor YadA, Ail a small (17 kDa) outer membrane protein of *Y. enterocolitica* serotype O:8, may also influence the susceptibility of *Y. enterocolitica* to complement-mediated killing [30], [31]. Genomic analysis of strains from two subspecies of *Y. enterocolitica*, subsp. *palearctica* 105.5R(r) and subsp. *enterocolitica* 8081 [32], [33], revealed the presence of two homologs of *ail*: one encoded by the *ail* gene and the other by the gene *ompX*. To identify these genes in the genome of strain Ye9, DNA fragments of 976 bp for *ail* (primers aA1 and aA976) and 883 bp for *ompX* (primers oX1 and oX883) were amplified by PCR and sequenced. Amino acid sequences were deduced from these fragments and multiple sequence alignment of Ail and OmpX from Ye9, *Y. enterocolitica* subsp. *enterocolitica* 8081, *Y. pestis* KIM [34] and *Enterobacter cloacae* subsp. *cloacae* ATCC 13047 [35] was performed (data not shown). The alignment of Ail and OmpX from strain Ye9 revealed 38% identity. OmpX from Ye9 displayed the highest identity (100%) to OmpX from *Y. enterocolitica* subsp. *enterocolitica* 8081, 90% identity to OmpX from *Y. pestis* KIM and 66% to OmpX from *Enterobacter cloacae* subsp. *cloacae* ATCC 13047. In comparison, Ail from Ye9 was 94% identical to Ail from *Y. enterocolitica* subsp. *enterocolitica* 8081 and 74% identical to Ail from *Y. pestis* KIM.

**Figure 4:**
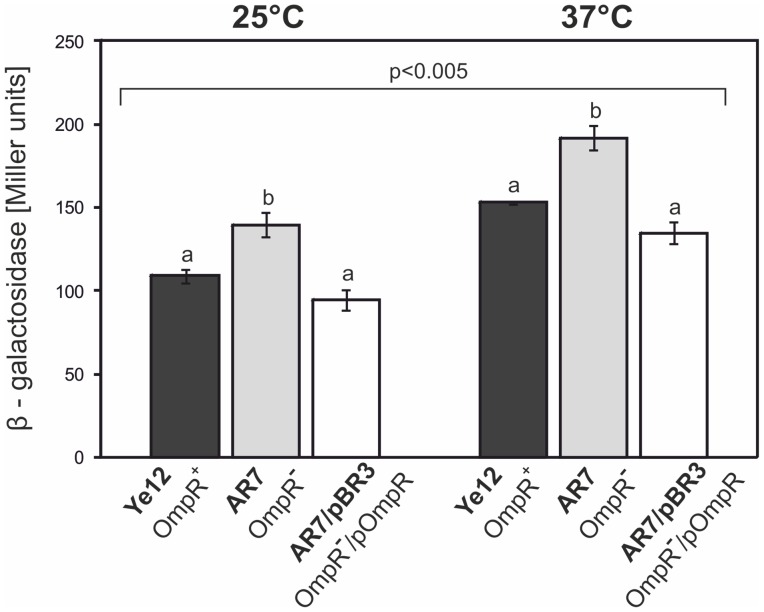
Effect of the OmpR protein on *ail* promoter activity in *Y. enterocolitica* O:9 strains. Strains Ye12 (OmpR^+^) and AR7 (OmpR^−^), representing the respective derivatives of Ye9 and AR4, harboring a transcriptional reporter *ail-lacZ* fusion, and strain AR7 with plasmid pBR3 expressing OmpR, were grown at 25 or 37°C in LB medium. The activity of β-galactosidase was assayed to evaluate *ail* expression. Values represent mean β-galactosidase activities, expressed in Miller units ± SD, from three independent experiments. Different letters (a, b) above the columns indicate statistically significant differences (P<0.005, covariance with Tukey correction).

To investigate the influence of OmpR on the expression of the *ail* gene, the suicide plasmid pFUSE carrying a promoterless *lacZYA*' operon was applied for insertional mutagenesis and the generation of chromosomal transcriptional fusions of the *ail* promoter with the *lacZ* reporter gene. The *ail* mutants were constructed in strains Ye9 (Nal^R^) and AR4, generating strains Ye12 and AR7, respectively. The structure of the mutated *ail* locus in both mutant strains was confirmed by PCR with primers FaA22 and lacZ3 (data not shown). To analyze whether the transcription of *ail* is OmpR-dependent and temperature regulated, β-galactosidase activity was assayed in strains Ye12 and AR7 grown at 25 and 37°C (Fig. **4**). The activity of the *ail* promoter (expressed by β-galactosidase activity) in both strains was higher at 37°C than at 25°C, indicating that transcription of this gene is subject to temperature regulation (P<0.005). The lack of OmpR in strain AR7 increased *ail* expression slightly at both temperatures, a change that was statistically significant compared to the level of expression in strain Ye12 (P<0.002), suggesting that OmpR maybe involved in regulating this gene, but plays no part in the response to changes in temperature. To confirm the effect of OmpR on the regulation of *ail* expression, a copy of the *ompR* gene in plasmid pBR3 (*ompR* cloned into medium-copy-number vector pBBR1 MCS-3) [18] was introduced into strain AR7. The wild-type phenotype was restored by the introduction of pBR3 (Fig. **4**), but not by the vector alone (data not shown). The β-galactosidase activity in strain AR7/pBR3 was decreased compared to AR7 and it was not significantly different from the activity in strain Ye12. These results implied that OmpR might directly or indirectly play a role in the negative regulation of *ail*, but further studies are required to precisely establish the function of OmpR.

### Serum-sensitivity phenotype of the *ail* mutant of *Y. enterocolitica* strain Ye9

**Figure 5:**
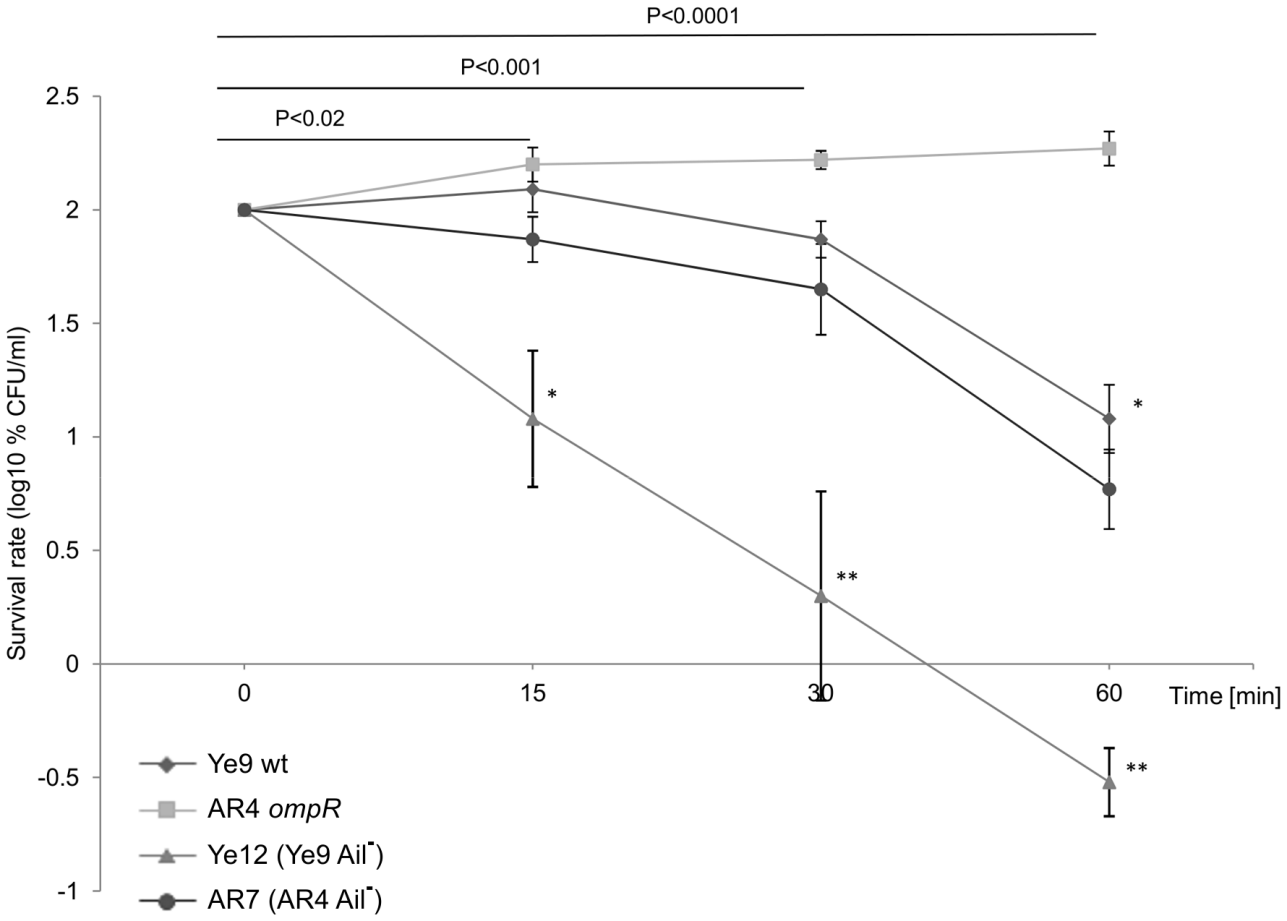
The susceptibility of *Y. enterocolitica* O:9 strains with and without protein Ail to normal human serum. Strains Ye9 (wt), Ye12 (Ye9 Ail^−^), AR4 (OmpR^−^) and AR7 (AR4 Ail^−^) were grown to exponential phase at 37°C and incubated with 50% NHS at 37°C. Aliquots removed at 0, 15, 30 and 60 min were plated for viability counts (CFU). The numbers of CFU/ml were normalized to the T_0_ values as a percentage and then converted to log_10_ to show the survival rate of the bacteria in NHS. Data are the means ±SD from three independent experiments. The asterisks indicate statistically significant differences between Ye9 and AR4 and their isogenic *ail* mutant Ye12 (Ail^−^) and AR7 (Ail^−^) (* P<0.001, ** P<0.0001, by one way ANOVA with Tukey's comparison post-test).

To determine the role of Ail in the serum resistance of *Y. enterocolitica*, the kinetics of serum killing of the Ye9 strain lacking protein Ail (strain Ye12) was analyzed (Fig. **5**). While Ye9 with active Ail exhibited moderate resistance to the bactericidal action of human serum and demonstrated 12% survival after 60-min incubation at 37°C, the introduction of the *ail* mutation rendered the cells more susceptible to complement-dependent killing (P<0.0001). Mutant strain Ye12 (Ail^−^) showed high serum susceptibility with a survival rate of 0.3% after 60-min incubation (Fig. **5**). Strain AR7, the *ail* knockout mutant of AR4, showed a slight decrease in serum resistance compared with its parent AR4 (P<0.001). Thus, the loss of protein Ail in both strains Ye9 and AR4 resulted in significantly decreased serum resistance at 37°C. Taken together, these data confirmed the known role of Ail in the serum resistance of *Y. enterocolitica* strains at 37°C and that the lack of Ail has a less drastic effect on serum resistance than the absence of YadA. Moreover, the comparable changes in serum resistance due to the lack of Ail observed in strains Ye9 and AR4, suggest that the serum resistance phenotype of the *ompR* mutant is not correlated with higher Ail activity in this strain.

### OmpR-dependent *ompX* expression in *Y. enterocolitica* Ye9

**Figure 6:**
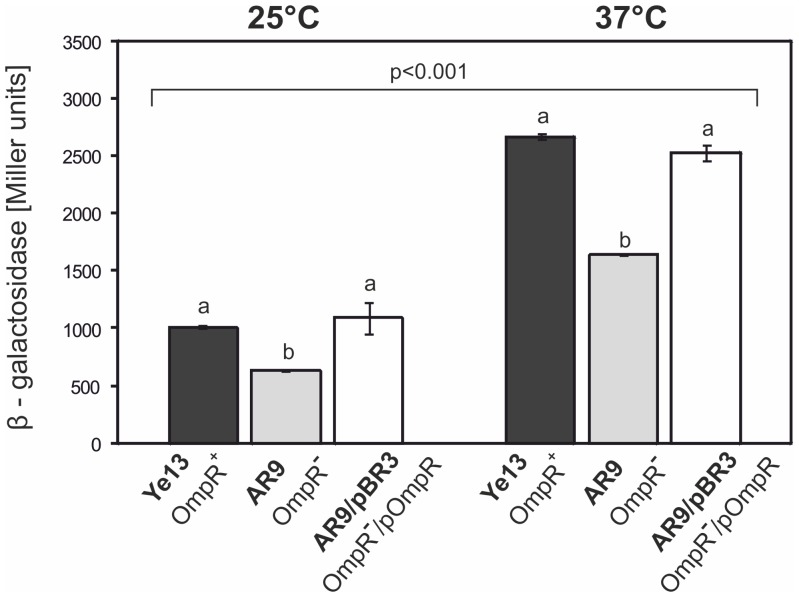
Effect of the OmpR protein on *ompX* promoter activity in *Y. enterocolitica* O:9 strains. The expression of *ompX* was measured by the activity of transcriptional reporter *ompX-lacZ* fusions. The strains Ye13, AR9 and AR9 with plasmid pBR3 expressing OmpR, were grown at temperatures of 25 or 37°C in LB medium and β-galactosidase activity was assayed to evaluate expression. Values represent the mean β-galactosidase activities expressed in Miller units ± SD from three independent experiments. Different letters (a, b) above the columns indicate statistically significant differences (p<0.05, covariance with Tukey correction).

Given the influence of the OmpR regulatory protein on *ail* gene expression, we examined OmpR-dependent regulation of the expression of the *ail* homolog *ompX*. Chromosomal transcriptional fusions of the *ompX* promoter with the *lacZ* reporter gene were constructed in strains Ye9 (Nal^R^) and AR4, yielding strains Ye13 and AR9, respectively. The structure of the mutated *ompX* locus in both mutant strains was confirmed by PCR with primers FoX175 and lacZ3 (data not shown). To analyze *ompX* transcription in strains Ye13 (OmpR^+^) and AR9 (OmpR^−^), the β-galactosidase activity in cells grown at 25 and 37°C was measured. As shown in Fig. **6**, a higher level of *ompX* transcription was observed at 37°C than at 25°C in both tested strains (P<0.001) In addition, the activity of the *ompX* promoter was significantly higher in Ye13 than in AR9 at both temperatures (P<0.05). The lack of the OmpR protein in AR9 resulted in a 1.6-fold decrease in β-galactosidase activity at 37°C, indicating a positive role for OmpR in *ompX* expression. Complementation by plasmid pBR3, which carries the active *ompR* allele, restored wild-type *ompX* expression in the *ompR* mutant AR9. The β-galactosidase activity in strain AR9/pBR3 was increased compared to AR9 and it was not significantly different from that in strain Ye13 (P>0.05) (Fig. **6**). Introduction of the vector alone (pBBR1 MCS-3) into AR9 had no effect on *ompX* regulation (data not shown).

### Serum-sensitivity phenotype of the *ompX* mutant of *Y. enterocolitica* strain Ye9

**Figure 7:**
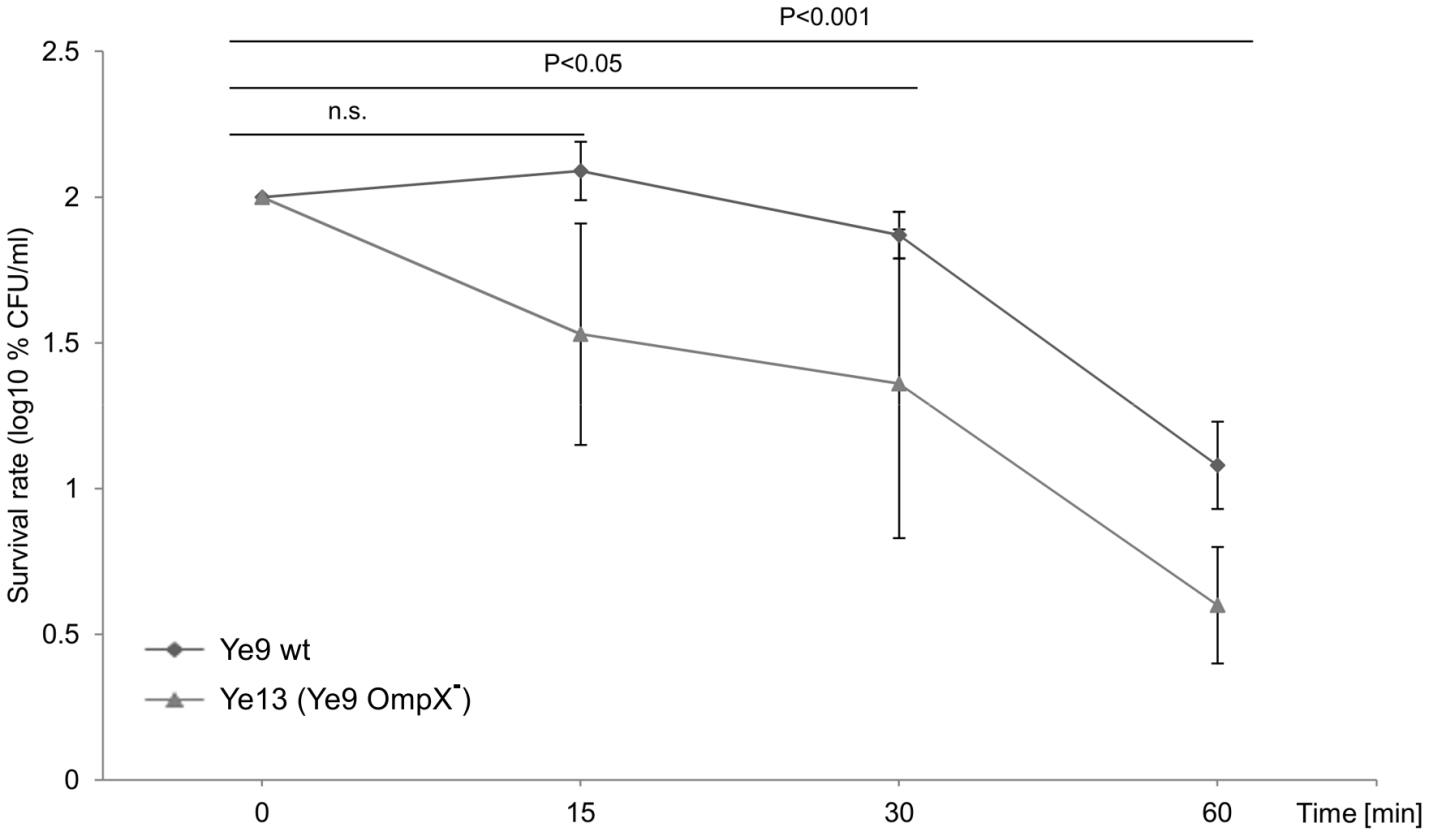
The susceptibility of *Y. enterocolitica* O:9 strains with and without protein OmpX to normal human serum. Strains Ye9 (wt) and Ye13 (Ye9 OmpX^−^) were grown to exponential phase at 37°C and incubated with 50% NHS at 37°C. Aliquots removed at 0, 15, 30 and 60 min were plated for viability counts (CFU). The numbers of CFU/ml were normalized to the T_0_ values as a percentage and then converted to log_10_ to show the survival rate of the bacteria in NHS. Data are the means ±SD from three independent experiments. The survival of both strains decreased in NHS during incubation and the differences between strains were not statistically significant (one way ANOVA with Tukey's comparison post-test).

To determine the influence of OmpX on serum resistance of *Y. enterocolitica*, the *ompX* insertion mutant of strain Ye9 (Ye13, OmpR^+^, OmpX^−^) was tested for its ability to survive serum killing at 37°C. This experiment revealed that the loss of OmpX did not significantly decrease resistance to complement-dependent killing (P>0.05) (Fig. **7**). Mutant strain Ye13 still exhibited moderate resistance to the bactericidal action of human serum and demonstrated 4% survival following incubation with 50% NHS at 37°C for 60 min. These data indicated that OmpX, a homolog of Ail, does not function in protecting the Ye9 cells from the harmful effects of human serum and may play an alternative role in the *Y. enterocolitica* outer membrane. Although not specifically examined, it was thought very unlikely that the lack of functional OmpX in the *ompR* mutant (with already decreased levels of *ompX* expression) would influence the serum resistance of this strain (AR4).

### Role of OmpC in serum resistance of *Y. enterocolitica* Ye9

**Figure 8:**
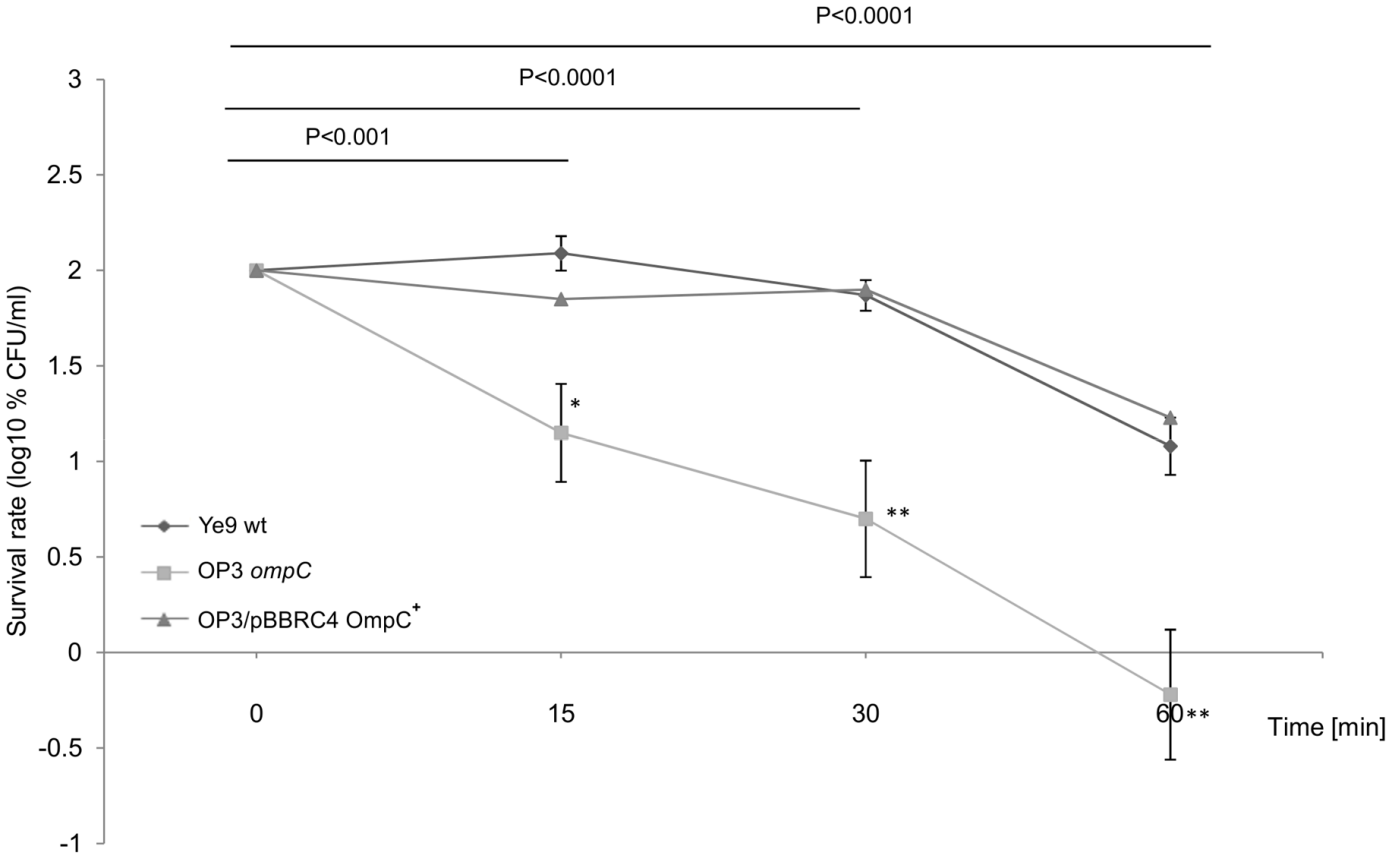
The susceptibility of *Y. enterocolitica* O:9 strains with and without protein OmpC to normal human serum. Strains Ye9 (wt), OP3 (Ye9 OmpC^−^) and OP3/pBBRC4 (*ompC* mutant OP3 with plasmid pBBRC4 OmpC^+^) were grown to exponential phase at 37°C and incubated with 50% NHS at 37°C. Aliquots removed at 0, 15, 30 and 60 min were plated for viability counts (CFU). The numbers of CFU/ml were normalized to the T_0_ values as a percentage and then converted to log_10_ to show the survival rate of the bacteria in NHS. Data are the means ±SD from three independent experiments, except for strain OP3 with plasmid pBBRC4 (performed only once). The asterisks indicate statistically significant differences between Ye9 and its *ompC* mutant OP3 (* P<0.001, ** P<0.0001, by one way ANOVA with Tukey's comparison post-test).

Our previous studies with *Y. enterocolitica* strains lacking OmpF, OmpC and both of these porins revealed their role in determining the permeability of the outer membrane to antibiotics including β-lactams [14]. We have also demonstrated that *Y. enterocolitica* OmpC (YompC) may function as an important virulence factor during bacterial infection [12]. To investigate the role of OmpC in the serum susceptibility of *Y. enterocolitica* Ye9, a strain carrying an *ompC* knockout mutation, named OP3 [12], was examined in serum survival assays. OP3 (OmpC^−^) and its parent strain Ye9 (OmpC^+^) showed significant differences in their sensitivity to complement-mediated killing (Fig. **8**). The survival of OP3 was reduced to approximately 0.6% after incubation with 50% NHS for 60 min, demonstrating a serum-sensitive phenotype (P<0.0001). The loss of OmpC decreased the serum resistance of Ye9 by ∼20-fold and the introduction of plasmid pBBRC4 [12], carrying an active *ompC* allele, into OP3 (strain OP3/pBBRC4), restored the wild-type serum resistance phenotype. Thus, the presence of OmpC in the OM appears to confer serum resistance to *Y. enterocolitica* strain Ye9. Despite this interesting finding, these results argue against a role for OmpC in the serum resistance phenotype of the *ompR* mutant AR4, which lacks this porin.

### Role of FlhDC in serum-resistance of *Y. enterocolitica* strains

Strain AR4, the *ompR* mutant of *Y. enterocolitica* Ye9, was previously characterized as non-motile at 25 and 37°C due to significantly reduced expression of *flhDC*, encoding the master activator of the flagellar type III secretion apparatus and flagella synthesis FlhDC [18], [36]. As for all strains of *Y. enterocolitica*, the motility of wild-type strain Ye9 is arrested at 37°C due to inhibition of the synthesis of the alternative sigma factor FliA controlling the expression of class III genes of the flagellar regulon, including those coding for flagellin proteins [37], [38]. Despite the lack of flagella, the flagellar type III secretion apparatus is still present [39], [40]. The expression of *flhDC* is under the positive control of the OmpR regulatory protein and the non-motile phenotype of the OmpR-deficient strain AR4 can be restored to the wild-type by complementing the mutation with a copy of the *ompR* gene on plasmid pBR3 [18], [19]. Moreover, this phenotype can be suppressed by FlhDC expressed in *trans* in *ompR* mutant cells (our unpublished observation).

**Figure 9:**
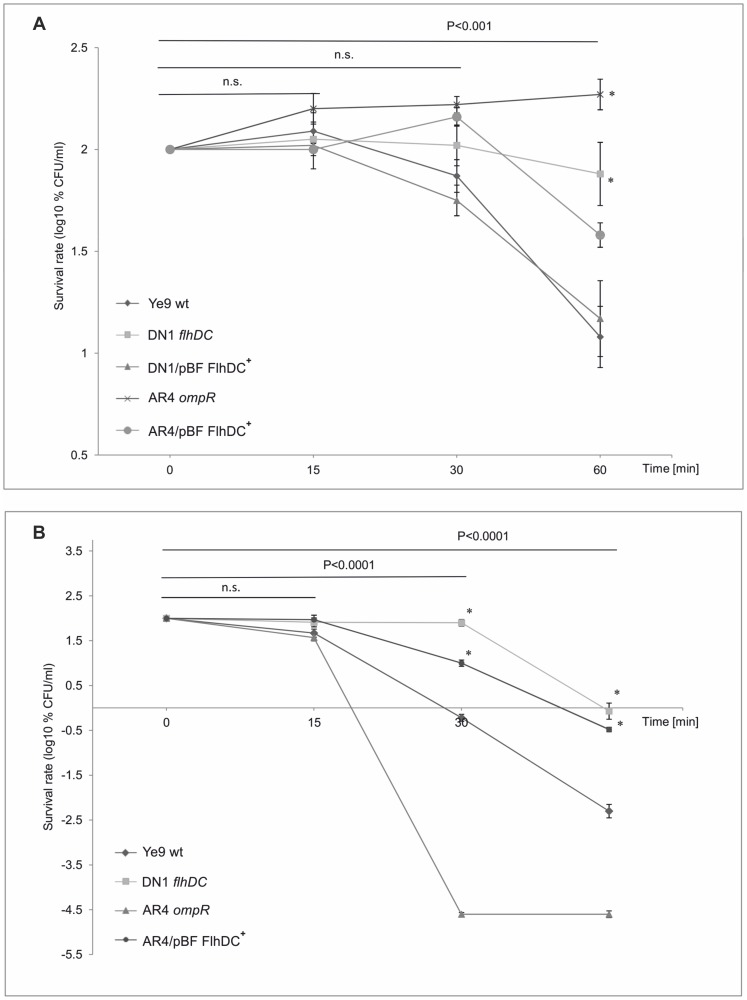
The susceptibility of *Y. enterocolitica* O:9 strains with and without protein FlhDC to normal human serum. **A**. Strains Ye9 (wt), AR4 (*ompR*), DN1 (*flhDC*) and both AR4 and DN1 with plasmid pBF carrying the wild-type copy of *flhDC* were grown to exponential phase at 37°C and incubated with 50% NHS at 37°C. Aliquots removed at 0, 15, 30 and 60 min were plated for viability counts (CFU). The numbers of CFU/ml were normalized to the T_0_ values as a percentage and then converted to log_10_ to show the survival rate of the bacteria in NHS. Data are the means ±SD from three independent experiments. The asterisks indicate statistically significant differences between strains with and without plasmid pBF (* P<0.001, by two way ANOVA with Tukey's comparison post-test). **B**. Survival of Ye9 (wt), DN1 (*flhDC*), AR4 (*ompR*) and AR4/pBF (*ompR* mutant AR4 with plasmid pBF FlhDC^+^) strains grown at 25°C in 50% NHS, determined as described above. Data are the means ±SD from three independent experiments. The asterisks indicate statistically significant differences between strains DN1 and AR4/pBF, and their parents, Ye9 and AR4, respectively (* P<0.001, ** P<0.0001, by one way ANOVA with Tukey's comparison post-test).

Based on the hypothesis that the high serum resistance of strain AR4 might be partly due to the downregulation of *flhDC* expression, we decided to evaluate the contribution of FlhDC to the sensitivity of *Y. enterocolitica* Ye9 to complement-mediated killing. Strain DN1, carrying the mutated *flhDC* locus [18], was examined in serum survival assays, as described above (Fig. **9A**). The results demonstrated that DN1 (FlhDC^−^) grown at 37°C displayed high-level serum resistance. After 60-min incubation in 50% NHS, 77% survival was observed, which is significantly (∼6.5-fold) higher than the survival rate of wild-type strain Ye9 (P<0.0001). Strain DN1 complemented with plasmid pBF, carrying the promoter-less *flhDC* operon cloned under the control of the exogenous p*_lac_* promoter of pBBR1 MCS-3, showed a decrease in serum resistance almost to the wild-type level (P<0.001). These results demonstrated the biological relevance of the loss of FlhDC to serum survival. Moreover, they suggested that the serum-resistant phenotype exhibited by the *ompR* mutant AR4 at 37°C is only partly the result of down-regulation of *flhDC* expression, since mutant DN1 displayed ∼2-fold lower serum resistance than *ompR* mutant AR4. To further support this hypothesis, plasmid pBF was introduced into strain AR4. A clear difference in serum resistance was seen between strain AR4/pBF, expressing wild-type *flhDC* in *trans*, and the parent AR4 (P<0.0001) (Fig. **9A**). The presence of the *flhDC* operon in multicopy in the AR4 strain caused a decrease in serum resistance of approximately 5-fold following 60-min incubation at 37°C in 50% NHS (P<0.001). However, the serum resistance did not return to the wild-type level and strain AR4/pBF was still ∼4-fold more resistant than Ye9 (P<0.001). This result confirmed that the serum resistance phenotype of the *ompR* mutant is partly due to a reduction in the level of FlhDC. The differences in serum susceptibility between the wild-type strain Ye9 and its derivative lacking FlhDC (DN1), both grown at 25°C, were studied further (Fig. **9B**). While the serum resistance of both strains was still very low (due to the fundamental regulatory mechanism decreasing YadA expression at low temperature), strain DN1 exhibited an increase in serum survival of about 200-fold compared with Ye9 after 60-min incubation in NHS (P<0.001). This result confirmed that the loss of FlhDC can promote serum survival of strain Ye9, although the role of FlhDC is clearly visible at low temperature, suggesting that an additional temperature-dependent regulatory factor/mechanism is involved. In addition, we investigated the serum survival of *ompR* mutant AR4 grown at 25°C, without or with wild-type *flhDC* expressed in *trans* (AR4/pBF) (Fig. **9B**). Surprisingly, the serum survival of strain AR4/pBF increased compared with that of the highly susceptible parent strain AR4 when grown at 25°C (in contrast to the situation at 37°C), suggesting the existence of some complex temperature-dependent feature of serum resistance in AR4.

### Lack of OmpR modifies the LPS O-antigen profile

**Figure 10:**
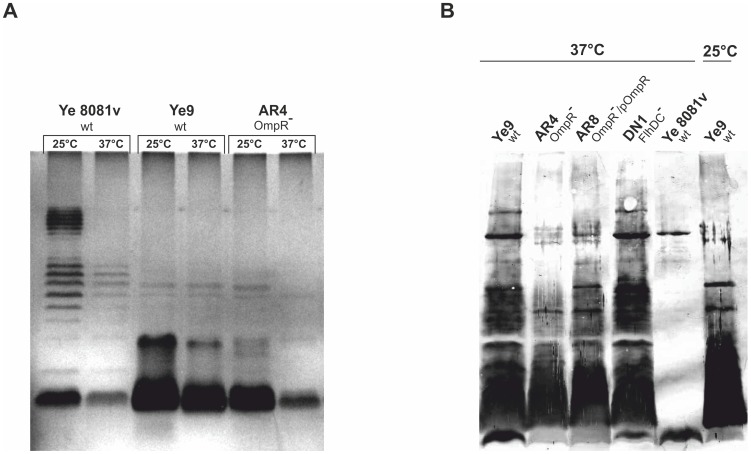
Analysis of LPS of *Y. enterocolitica* O:9 strains. **A**. The bacteria were grown in LB medium at 25 or 37°C to an OD_600_ of 1.2. LPS was extracted by the phenol-water method and subjected to electrophoresis in 15% SDS-polyacrylamide gels. The LPS was visualized by silver staining. The gel shown is representative of the results of an experiment performed twice. **B**. Whole-cell lysates of different strains grown at 25 or 37°C to an OD_600_ of 0.3 were separated by SDS-PAGE and immunoblot analysis was performed using polyclonal antiserum specific for *Y. enterocolitica* serotype O:9 LPS.

The above observations posed two questions: (1) how does the *flhDC* mutation influence serum susceptibility, and (2) what other changes in the *ompR* mutant might be responsible for its extremely high serum resistance at 37°C? In addition, our initial experiments examining sensitivity to NHS revealed that temperature has a greater impact on the serum resistance phenotype of the *ompR* mutant than that of the wild-type Ye9. This observation led to the hypothesis that a temperature-regulated surface structure of *Y. enterocolitica* might be changed in the mutant cells to affect serum resistance. The strongly temperature-dependent part of the outer membrane is the lipopolysaccharide (LPS). LPS, a major component of the outer leaflet of the outer membrane, is composed, in the complete (S-form), of three major domains: lipid A, the core (inner and outer parts) and the O-specific polysaccharide (O-antigen) [41]. To examine whether LPS content/status is altered in the *ompR* mutant cells, LPS was extracted from cells of strains Ye9 and AR4 and analyzed by SDS-PAGE (Fig. **10A**). The LPS structure and the effect of growth temperature on the LPS status was also analyzed in *Y. enterocolitica* strain 8081 serotype O:8, examined earlier for its YadA content (Fig. **2B**). This SDS-PAGE analysis demonstrated that indeed temperature affects the LPS status of cells of *Y. enterocolitica* Ye9 (serotype O:9). At 37°C, strain Ye9 expressed less O-antigen/LPS than at 25°C. The same thermoregulation of LPS was observed in strain 8081 (serotype O:8). However, the LPS migration patterns of strain Ye9 and strain 8081 were different. The serotype O:8 strain displayed the classical ladder on SDS-PAGE, corresponding to the smooth form of LPS. In contrast, the LPS of Ye9 resembled the semi-rough profile. The LPS migration pattern of *ompR* mutant strain AR4 was the same as that of parental strain Ye9 and the temperature regulation phenotype of these two strains was similar. However, when grown at either 25 or 37°C, strain AR4 exhibited significantly lower levels of LPS than strain Ye9. Thus, the *ompR* mutation altered the overall LPS content. To rule out the possibility that the LPS isolation procedure was responsible for the apparent differences in LPS content, the LPS of the separate *Y. enterocolitica* strains was examined by immunoblot analysis of whole-cell extracts using a polyclonal antiserum specific for *Y. enterocolitica* serotype O:9 LPS (Fig. **10B**). Comparison of the intensity of the immunoblot signals confirmed that strain Ye9 grown at 25°C produced less LPS than at 37°C. In addition, the *ompR* mutant strain AR4 had a significantly lower LPS content than Ye9 at 37°C. To confirm the role of OmpR in the regulation of LPS production, strain AR8, i.e. AR4 transformed with plasmid pHR4 carrying the *ompR* gene, was analyzed. Complementation of the *ompR* mutation by pHR4 almost restored the wild-type level of LPS in AR4. Furthermore, no difference in the intensity of the LPS signal of the *flhDC* mutant strain DN1 and the wild-type strain Ye9 was observed when both were grown at 37°C (Fig. **10B**). Together, these data suggest that two independent factors might influence the serum resistance of the *Y. enterocolitica ompR* mutant.

## Discussion

We have previously demonstrated that the OmpR protein of *Y. enterocolitica* strain Ye9 (serotype O:9) controls the transcription of several genes that may confer an advantage in a particular ecological niche [15], [17], [18], [19]. In the present study, we attempted to further characterize the functions of OmpR by examining the ability of the *ompR* mutant strain AR4 to survive complement-dependent killing by human serum: the first-line cellular defense against bacterial pathogens [21]. Initially we showed that the *Y. enterocolitica* wild-type strain Ye9 has moderate resistance to the bactericidal activity of human serum compared with the high resistance to NHS of serotypes O:8 and O:3 [25], [42], [43]. The same moderate resistance phenotype has been described previously for other clinical *Y. enterocolitica* serotype O:9 strains [25] and probably reflects the host range of this serotype, which infects animals and displays low virulence in humans [22]. The higher resistance of strain Ye9 to bactericidal killing by bovine serum (our unpublished observation) seems to support this suggestion. We then demonstrated that the *Y. enterocolitica* mutant AR4 (lacking OmpR protein) displays at least 15-fold higher resistance to NHS compared with the wild-type parent Ye9. This suggested that the OmpR regulator, the effector of a two-component transduction system that is activated (phosphorylated) in response to certain environmental signals, might alter factors that localize in the bacterial outer membrane to affect sensitivity to complement-mediated killing. We envisaged two possible scenarios that might account for this phenomenon. Our first hypothesis was connected with the role of OmpR in the regulation of OMPs known to directly influence the complement system, while the second was that OmpR indirectly influences OMPs and/or regulatory proteins involved in serum survival. We did not rule out the possibility that OmpR could affect serum resistance by modifying outer membrane structure and in this way modulate the activity of proteins responsible for this resistance. Our efforts to understand the role of OmpR in the serum susceptibility of *Y. enterocolitica* Ye9 revealed a complex regulatory network.

To gain some insight into the mechanism responsible for the high serum resistance of *Y. enterocolitica* strain AR4, we started by investigating OMPs and specifically YadA, the major player in serum resistance in *Y. enterocolitica*. YadA is an outer membrane protein encoded on the virulence plasmid pYV that is expressed mainly at 37°C [28]. This OMP plays a crucial role in protecting *Yersiniae* against the classical and alternative pathways of complement killing [24], [44], [45], [46], [47], [48], [49]. Thus, one possible explanation for the high serum resistance of the *ompR* mutant is that overexpression of YadA protects the mutant cells against the activity of complement. However, we did not observe significant differences in the YadA content in OMP samples isolated from *Y. enterocolitica* wild-type strain Ye9 and the *ompR* mutant AR4, when both were grown at 37°C. In fact, the most visible difference between the two OMP profiles was the presence of the major porins OmpF/C in Ye9 that were not detected in the outer membrane of strain AR4 at 25 or 37°C, indicating that their expression is OmpR-dependent. This phenotype is well known and has been described previously in *Y. enterocolitica* [17].

Although YadA is recognized as the most important serum resistance factor of *Y. enterocolitica*, the chromosomally-encoded Ail protein has also been found to confer resistance to complement-mediated killing in this enteropathogen [30]. Moreover, it has been shown that Ail cooperates with YadA in complement resistance [45], [50]. Ail from *Y. enterocolitica* is a small OMP belonging to the large Ail/OmpX/PagC/Rck/Lom family of outer membrane proteins that function at least in part to protect Gram-negative bacteria from complement-mediated killing [51], [52], [53], [54], [55]. Some Ail homologs have been identified in other pathogenic *Yersiniae*, i.e. *Y. pseudotuberculosis* and *Y. pestis* [56], [57], [58], [59]. In the course of this study, genes encoding two representatives of this family of OMPs were identified in the genome of *Y. enterocolitica* Ye9. They were designated according to their annotation in the 8081 genome as *ail* and *ompX*. The study of transcriptional gene fusions revealed that OmpR acts to negatively (*ail*) and positively (*ompX*) regulate the expression of these two homologous OMPs of *Y. enterocolitica* Ye9. Moreover, in contrast to *ail* from *Y. enterocolitica* 8081, which is expressed mainly at 37°C [31], *ail* expression in Ye9 was not subject to such obvious thermoregulation and occurred at both temperatures, albeit at a higher level at 37°C. Temperature-independent expression of *ail* was also recently described in *Y. pestis* [57], [58]. A role for OmpR in the positive regulation of *Y. pestis ompX* expression was demonstrated previously [60].

To define the role of OmpR in the serum sensitivity of *Y. enterocolitica* Ye9 and also the precise function of the proteins YadA, Ail and OmpX in this phenomenon, mutant strains were constructed and examined in serum susceptibility assays at 25 and 37°C. Since the biological role of the OmpC porin of *Y. enterocolitica* in serum resistance has not previously been studied, an *ompC* null mutant was also included in this analysis. Our findings demonstrated that the fundamental, thermo-regulated serum resistance phenotype of strain Ye9 correlates with the expression of YadA encoded by plasmid pYV. Although OmpR is not involved in this primary thermoregulation, we observed the dramatic impact of the absence of the YadA protein on the bactericidal effect of serum in the *ompR* mutant background. The lack of YadA reduced the high serum resistance phenotype of the *ompR* mutant by ∼10,000-fold (only 200-fold in Ye9 wt). As the level of YadA in the OM of mutant AR4 was not raised significantly, we speculate that the *ompR* mutation might potentiate the function of YadA. Tests on the *ail* and *ompX* mutants to measure survival percentages and kinetic killing provided evidence that Ail confers serum resistance to the Ye9 strain, but its effect on the high serum resistance of *ompR* mutant cells was not visible. In contrast to Ail, the OmpX homolog had no influence on complement-dependent killing of the Ye9 cells; thus this OMP probably plays a separate role in the physiology of *Y. enterocolitica*. Our examination of the functional properties of the OmpC porin revealed that the presence of this protein in the OM confers serum resistance in wild-type *Y. enterocolitica* Ye9. The opposite effect, i.e. increased serum survival of an *ompC* mutant, has been described recently in *E. coli* [61]. In addition, studies on the outer membrane porins OmpK35 and OmpK36 of *Klebsiella pneumoniae* (homologs of *E. coli* OmpF and OmpC) demonstrated no correlation between their presence and susceptibility to the bactericidal effect of serum [62]. Therefore, it seems that the role of porins in serum resistance depends on the bacterial species. Despite this interesting finding, our results argue against a role for OmpC in the serum resistance of the *Y. enterocolitica ompR* mutant AR4 (lacking both OmpC/OmpF porins).

One of the most intriguing findings of the present study was the impact of FlhDC on serum resistance of *Y. enterocolitica* Ye9 and the *ompR* mutant strain AR4. We recently described the positive role of OmpR in the regulation of *flhDC*, which encodes the master flagellar regulator FlhDC [18]. FlhDC activates the transcription of a set of structural genes encoding the flagellar type III secretion system and regulatory genes, i.e. the alternative sigma factor FliA and its antagonist, the anti-sigma factor FlgM [36], [38], [63]. Serum killing tests showed that a decreased level of FlhDC in the *ompR* mutant is partly responsible for its serum resistance. This was confirmed by the demonstration that this phenotype can be suppressed by constitutive multicopy expression of *flhDC* in *trans*. This possibility was strengthened by the observation that the loss of FlhDC enhanced the survival of wild-type cells in NHS. Based on these findings, we hypothesize that the decrease in FlhDC might influence OM structure by causing changes, for example, in the flagellar type III secretion apparatus content, resulting in greater exposure of YadA, and thus conferring serum resistance to the *ompR* mutant strain of *Y. enterocolitica*. However, since the loss of FlhDC was more beneficial to the survival of wild-type cells in NHS at 25°C compared to 37°C, we do not rule out the possibility that the serum susceptibility governed by FlhDC might be partly related to the activity of additional temperature-regulated effectors. The best candidate for a protein with such a regulatory function is the alternative sigma factor FliA, which is positively regulated by FlhDC. The expression of FliA is thermoregulated (repressed at 37°C) independently of FlhDC and exerts a negative effect on the expression of VirF, the transcriptional activator of *yadA* [28], [38]. Although we have found no differences in the level of YadA in the OM of *flhDC* mutant cells (our unpublished observation), this aspect of FlhDC regulation requires further investigation.

Another possible cause of the serum-resistant phenotype of the *ompR* mutant that was considered, is based on the putative role of LPS in serum resistance. We found that the loss of OmpR correlated with a reduced LPS content in the outer membrane of bacteria grown at 25 and 37°C. However, such a correlation was not observed in the case of the *flhDC* mutant, indicating that FlhDC-mediated serum resistance occurs independently of LPS. Our results also confirmed the effect of growth temperature on the LPS structure of strain Ye9 (serotype O:9), a phenomenon described previously in serotypes O:8, O:3 and O:9 [24], [25], [42]. In addition, analysis of the LPS of strain Ye9 demonstrated that its profile is different from that of serotype O:8 strain 8081, which reflects the unique structural features of serotype O:9 LPS [43], [64], [65], [66]. Based on our analysis of the OM proteins and LPS content of *Y. enterocolitica* Ye9 and its mutants, we suggest that, apart from the role of FlhDC, changes in the outer membrane LPS content could influence the activity/exposure of YadA in the *ompR* mutant. Such a correlation has previously been demonstrated for other *Yersinia* virulence factors including Ail [24], [42], [47], [58], [67] and invasin protein [68]. It is tempting to speculate that the *Y. enterocolitica* OmpR regulator can modulate FlhDC and LPS expression in response to different environmental signals and control the function of virulence factors required in certain host tissues. There may be an as yet uncharacterized regulatory network centered on OmpR that coordinates the expression of FlhDC and LPS synthesis, thus modulating OM structure. The OmpR-dependent interrelationship of surface-anchored cellular components of *Y. enterocolitica* could permit switching between distinct niches within and outside the host body, where bacteria face a constant adaptive challenge. The gain of fitness provided by the EnvZ/OmpR TCS might result primarily from its role in serum resistance.

## Materials and Methods

### Normal human serum

Normal Human Serum (NHS) was obtained from 30 healthy donors at the Regional Centre of Transfusion Medicine and Blood Bank, Wroclaw, Poland. This was conducted according to the principles expressed in the Declaration of Helsinki and was approved by the author' institutional review board (El¿bieta Klausa). Experiments were performed with NHS samples taken from individual donors and mixted together. Blood samples were taken into aseptic dry tubes or tubes with anticoagulant. After collection, the samples were stored at room temperature (RT, 22°C±2°C) for 1 hour to allow separation. The serum samples were then centrifuged for 10 min at 3500 rpm to pellet cells and platelets. Only serum samples lacking hemolysis and with negative test results for HIV, HCV and *Treponema pallidum* antibodies, a negative HBs antigen test, and negative viral genome screen (HIV RNA, HBV DNA, HCV RNA) were used. The C3 concentration in the mixed serum, quantified by radial immunodiffusion (Human Complement C3 & C4 ‘Nl’ Bindarid™ Radial Immunodiffusion Kit; The Binding Site Group Ltd.), was 1330 mg/l. The serum was frozen in 0.5-ml and 1-ml aliquots at −70°C for periods no longer than 2 months. The required volume of serum was thawed immediately before use and each portion was used only once.

### Bacterial strains, media and growth conditions

The strains used in this study are listed in Table **1**. *E. coli* and *Yersinia* strains were routinely grown under aerobic conditions at 25 or 37°C in LB (Luria-Bertani) and BHI (Brain Heart Infusion) broth or on LB agar plates. The antibiotics used for bacterial selection were as follows: kanamycin (Km) – 50 µg ml^−1^, chloramphenicol (Cm) – 25 µg ml^−1^, nalidixic acid (Nal) – 30 µg ml^−1^, tetracycline (Tet) – 12.5 µg ml^−1^.

**Table 1:** Strains and plasmids used in this study.

Strains and plasmids	Description	Reference or source
***Y. enterocolitica*** ** O:8**		
Ye 8081v	pYV^+^ patient isolate, wild-type	[75]
***Y. enterocolitica*** ** O:9**		
Ye9	pYV^+^, wild-type	Clinical isolate, laboratory collection
Ye9c	pYV-cured derivative of Ye9	This work
Ye9N	Ye9, Nal^R^	[17]
Ye12	Ye9N, *ail*::*lacZYA*, Nal^R^, Cm^R^	This work
Ye13	Ye9N, *ompX*::*lacZYA*, Nal^R^, Cm^R^	This work
DN1	Ye9N, *flhDC*::pDS132, Nal^R^, Cm^R^	[18]
OP3	Ye9, Δ*ompC*, Nal^R^, Cm^R^	[12]
AR4	pYV^+^, Δ*ompR*::Km, Nal^R^, Km^R^	[15]
AR4c	pYV-cured derivative of AR4	This work
AR7	AR4, *ail*::*lacZYA*, Nal^R^, Km^R^, Cm^R^	This work
AR8	AR4, pHR4	[15]
AR9	AR4, *ompX*::*lacZYA*, Nal^R^, Km^R^, Cm^R^	This work
***E. coli***		
S17-1 λ*pir*	*pro thi recA hsdR514* (R^+^M^−^) *λpir* RP4 2-Tc::Mu-Kn::Tn7 (Tp^R^ Str^R^)	[76]
**Plasmids**		
pDrive	cloning vector, Amp^R^, Km^R^	Qiagen
pFUSE	suicide vector, derivative of pEP185.2 with promoterless *lacZYA* genes, Cm^R^	[77]
pFA	pFUSE with XbaI/SmaI fragment (706 bp) of *ail*, Cm^R^	This work
pFX	pFUSE with XbaI/SmaI fragment (400 bp) of *ompX*, Cm^R^	This work
pBBR1 MCS-3	cloning vector; Tet^R^	[78]
pBF	pBBR1 MCS-3 carrying PstI/XbaI fragment with entire coding sequence of *flhDC*, Tet^R^	This work
pHR4	pHSG 575 carrying entire coding sequence of *ompR*, Cm^R^	[15]
pBBRC4	pBBR1 MCS-2 carrying entire coding sequence of *ompC*, Km^R^	[12]
pBR3	pBBR1 MCS-3 carrying entire coding sequence of *ompR*, Tet^R^	[18]

### DNA manipulation and construction of plasmids

All DNA manipulations, including the polymerase chain reaction (PCR), restriction digests, ligation and transformation were performed according to standard procedures [69], [70]. Chromosomal and plasmid DNA were purified using the EURx Bacterial & Yeast Genomic DNA Purification Kit and Plasmid Miniprep DNA Purification Kit, respectively. Restriction enzymes were obtained from Thermo Scientific. Oligonucleotide primers used for PCR and sequencing were purchased from the DNA Sequencing and Oligonucleotide Synthesis Laboratory IBB PAN (Warsaw, Poland). PCR was routinely performed in 50-µl reaction mixtures for 28 cycles using *Taq* polymerase or Phusion High-Fidelity DNA polymerase (Thermo Scientific) according to the manufacturer's instructions. DNA fragments amplified by PCR were purified with a EURx PCR/DNA Clean-Up Purification Kit before and after restriction digestion. Sequencing reactions were performed by the DNA Sequencing and Oligonucleotide Synthesis Laboratory IBB PAN (Warsaw, Poland). Plasmids used in this study are listed in Table **1**, and primers in Table S3. Plasmids for single cross-over recombination were constructed by amplification of the *ail* and *ompX* genes from *Y. enterocolitica* Ye9 genomic DNA using primer pairs aA2X/aA706S and oX2X/oX400S, respectively, and then cloning these fragments into XbaI/SmaI-digested plasmid pFUSE. pBF was obtained by insertion of a PCR fragment (containing the *flhDC* operon), amplified from Ye9 genomic DNA with primers fDC1P/fDC1072X, into PstI/XbaI-digested plasmid pBBR1 MCS-3.

### Inactivation of *ail* and *ompX* genes and construction of chromosomal *lacZ* reporter fusions

The suicide vector pFUSE was used to create genomic insertions by homologous recombination. This plasmid contains the RK6 origin of replication and the RP4 origin of transfer, plus a promoterless *lacZYA*' operon for the construction of chromosomal *lacZ* reporter fusions. DNA fragments of the *ail* (706 bp, from position −403 to +303 relative to ATG codon) and *ompX* (400 bp, from position −23 to +377 relative to ATG codon) genes amplified by PCR were cloned into vector pDrive (Qiagen). The resulting constructs were digested with XbaI and SmaI, and the gene fragments subcloned into the XbaI/SmaI-restricted suicide vector pFUSE. The suicide vector constructs containing the *ail* and *ompX* fragments were named pFUSEA and pFUSEX, respectively. These plasmids were used to transform *E. coli* S17(λ*pir*) and a representative clone for each gene was mated with *Y. enterocolitica* strains. Transconjugants were selected on LB agar supplemented with Nal and Cm for Ye9N, and with Km and Cm for strain AR4 (Δ*ompR*::Km). Because pFUSE cannot replicate in *Y. enterocolitica* cells, all selected transconjugants carried the plasmid integrated into the chromosome. Homologous single-crossover recombination yielded strains carrying an insertional mutation in the *ail* or *ompX* genes and a chromosomal transcriptional fusion between the respective promoters (P_ail_ or P_ompX_) and the promoterless *lacZYA'* operon. One transconjugant of each type was examined for appropriate insertion of the suicide vector in the chromosome by PCR with one primer (FaA22 or Fox175), located upstream of the homologous region used for recombination and the other primer (lacZ3), within the *lacZ* gene. The constructed strains served as (i) the mutant for the particular gene and (ii) a strain carrying a single transcriptional fusion of the promoter with the *lacZ* reporter gene.

### Isolation of derivatives of *Y. enterocolitica* strains lacking plasmid pYV

Plasmidless derivatives of *Y. enterocolitica* strains were isolated following growth on BHI agar supplemented with 5 mM EGTA for 48 h at 37°C, as described previously [71]. Loss of plasmid pYV in spontaneously cured derivatives was verified by PCR using primers yH1 and yH517 designed to amplify pYV-encoded gene *yopH*. Clones were also shown to be cured by screening for plasmid DNA and autoagglutination tests (data not shown). The cured derivatives of strains Ye9 and AR4 were designated Ye9c and AR4c, respectively.

### β-Galactosidase assay

The activity of β-galactosidase in permeabilized cells was measured by the conversion of *o*-nitrophenyl-β-d-galactopyranoside into nitrophenol as described by Miller (1972). The activities were calculated as follows: 1000·(OD_420_-(1.75·OD_550_)·OD_600_^−1^Δt (min)^−1^vol (ml)^−1^.

### Outer membrane protein preparation and SDS-PAGE

The OM protein profiles of *Y. enterocolitica* strains were examined using bacteria grown in LB medium at 25 or 37°C. Briefly, a bacterial culture grown to an OD_600_ of 1.0 was centrifuged (12,000×g, 15 min), and the cell pellet resuspended in 4 ml of phosphate buffer (pH 7.5) containing 1 mM phenylmethylsulfonyl fluoride (PMSF). The cell suspension was sonicated on ice (six times for 15 s with 1-min breaks), and unbroken cells were removed by centrifugation for 3 min at 15,000×g. The supernatant was ultracentrifuged (45,000×g) for 45 min at 4°C and the pellet resuspended in a buffer containing 10 mM Tris (pH 8.0), 5 mM MgCl_2_, 2% sarcosine and 1 mM PMSF. The sample was incubated at RT for 20 min with frequent agitation and then ultracentrifugation was repeated. The pellet containing OMPs was resuspended in 50 µl of sample buffer (100 mM Tris-HCl pH 6.8, 2% SDS, 10% glycerol, 3% DTT, 0.001% bromophenol blue). Sample aliquots containing equal amounts of protein were heated at 95°C for 15 min and then subjected to SDS-polyacrylamide gel electrophoresis (SDS-PAGE) on 12% polyacrylamide gels [72]. The separated proteins were stained with Coomassie blue R.

### Tandem mass spectrometry (MS/MS) analysis

Selected protein bands observed on stained SDS-polyacrylamide gels were excised, digested with trypsin and subjected to liquid chromatography-tandem mass spectrometry (LC MS/MS) (IBB PAN, Warsaw, Poland).

### Isolation of LPS and analysis by SDS-PAGE

Lipopolysaccharide (LPS) was extracted from cells of *Y. enterocolitica* Ye9 (serotype O:9) and its derivative strains AR4 and AR8 grown in LB at 25 or 37°C. Extractions were optimal using 2 ml of culture at an OD_600_ of 1.2. LPS was prepared from the cells using an LPS Extraction Kit (iNtRON), which employs the phenol-water procedure. To obtain higher purity preparations of LPS, the samples were treated with proteinase K (A&A Biotechnology). LPS extracts were analyzed by discontinuous SDS-PAGE using the Laemmli buffer system [72]. Gel electrophoresis was performed in 1-mm slabs comprised of a 6% polyacrylamide stacking gel and 15% separating gel. Samples were applied to the slabs after heating at 100°C for 6 min. The running buffer was composed of 25 mM Tris, 192 mM glycine and 0.1% SDS. The electrophoretic separation was performed at constant power (1 W) using a Mini-Protean Tetra Cell apparatus (Bio-Rad). The separated LPS was visualized by silver staining using the procedure of Tsai and Frasch [73] with slight modifications. Banding patterns were visualized under white light and photographed using a GelDoc XR imaging system (Bio-Rad).

### Immunoblot analysis

Bacteria were grown to exponential phase in LB medium. Whole-cell lysates were prepared from 5 ml of culture adjusted to an OD_600_ of 0.15. The cells were collected by centrifugation for 15 min (1500×g) and the pellets resuspended in 100 µl of Laemmli sample buffer. For the verification of LPS expression, samples were digested with proteinase K prior to adding the sample buffer. The mixtures were heated at 95°C for 10 min and 10-µl aliquots were separated by SDS-PAGE on 8 or 12% polyacrylamide gels (to verify expression of YadA or LPS, respectively). The separated samples were transferred to nitrocellulose membrane using the semi-wet transfer method. Non-specific binding sites were blocked by bathing the membrane in 5% skimmed milk-PBS solution. Membranes were then incubated overnight at 4°C with polyclonal anti-YadA (a kind gift of J. Heesemann, Ludwig Maximilian University of Munich, Munich, Germany) or anti-LPS antisera (a kind gift of W. Rastawicki, National Institute of Hygiene, Warsaw, Poland), diluted 1∶1000. After three washes in PBS, the membranes were incubated with alkaline phosphatase-conjugated goat anti-rabbit immunoglobulins as the secondary antibody (dilution 1∶3000) for 1 h at RT. Following further washes in PBS, specific antibody binding was detected using the chromogenic substrate 5-Bromo-4-chloro-3-indolyl Phosphate/Nitroblue Tetrazolium (BCIP/NBT, Merck).

### NHS bactericidal assay

The bactericidal activity of NHS was determined as described previously [74] with slight modifications. Briefly, LB broth was inoculated to an OD_600_ of 0.1 with an overnight culture of the desired *Y. enterocolitica* strain. After incubation at 25 or 37°C in a water bath to achieve an OD_600_ of 0.3, the cells were collected by centrifugation (4000×g for 20 min at 4°C). The pellet was resuspended in 3 ml of physiological saline (0.9% NaCl) and then diluted in the same saline to produce a cell density of approximately 10^5^–10^6^ cells/ml. Aliquots of the cell suspension were mixed with an equal volume of NHS or heat-inactivated serum (HIS; incubated at 56°C for 30 min to inactivate complement) and these mixtures were incubated at 37°C for 0, 15, 30 and 60 min (T_0_, T_1/4_, T_1/2_ and T_1_, respectively) in a water bath with shaking at 200 rpm. Appropriate dilutions were then plated in duplicate or triplicate onto LB agar plates and incubated at 25°C for 48 h. The average number of colony-forming units per milliliter (CFU/ml) was calculated from the replicate plate counts. The survival rate was calculated as a percentage of the original cell number at T_0_ (100% survival). Strains with survival rates of >50% after 60-min incubation with serum were considered resistant, and those with survival rates of <1% were considered susceptible to the bactericidal action of NHS. Strains with survival rates of between 1 and 50% were considered to have intermediate resistance to NHS.

### Statistical analyses

The numbers of colony-forming units per milliliter (CFU/ml) from the bactericidal assays were converted to percentage survival values, where the starting inoculum at T_0_ was set at 100%, and values for the survival rates were expressed as log_10_ % CFU/ml. The data from each assay were compared by analysis of variance (repeated measures ANOVA). ANOVA was used to test the significant difference of decreases in survival or eradication of bacteria following incubation for 15, 30 and 60 min at 25 or 37°C in the presence of 50% NHS or HIS. Bacterial survival rates were compared using the planned comparison post-test of ANOVA. Regression and covariance analyses were used to examine differences in the activity of β-galactosidase (in Miller Units) between mutants of Ye9 (Nal^R^ resistant) and AR4, as well as for comparisons of the enzyme activity in cells grown at different temperatures. The P-values from these tests are shown in the text and the figures. p<0.05 indicates that the compared values are significantly different at a 95% confidence level.

## Supporting Information

Table S1 **Serum-resistance phenotypes and survival rates of Y. enterocolitica cells.**
(DOC)

Table S2 **Survival of bacterial cells in heat-inactivated serum (HIS).**
(DOC)

Table S3 **Oligonucleotide primers used for PCR and DNA sequencing in this study.**
(DOC)
