# Supplementary material for: Regulatory Protein OmpR Influences the Serum Resistance of Yersinia enterocolitica O:9 by Modifying the Structure of the Outer Membrane
Source: PLoS One. 2013 Nov 19;8(11):e79525. doi: 10.1371/journal.pone.0079525 (PMC3834241; doi:10.1371/journal.pone.0079525)
Supplement: Table S1 — Serum-resistance phenotypes and survival rates of Y. enterocolitica cells. (DOC) [file pone.0079525.s001.doc]

**Table S1. Serum-resistance phenotypes and survival rates of *Y. enterocolitica* cells at 37^o^C (unless indicated as 25^o^C). Data presented in Figs. 1, 3, 5, 7, 8, 9.**

| **Strain** | **Phenotype^a^** | **CFU^b^/ml** | | | | **Survival of cells at T_60_^d^ (%± SD)** | **Resistance phenotype^e^** |
| --- | --- | --- | --- | --- | --- | --- | --- |
|  |  | **T_0_^c^** | **T_15_** | **T_30_** | **T_60_** |  |  |
| Ye9 wt | YadA, Ail, OmpX, OmpC, FlhDC | 2.05 x 10^6^ | 2.56 x 10^6^ | 1.52 x 10^6^ | 2.36 x 10^5^ | 12 ± 4 | Intermediate |
| Ye9 wt (25^o^C) | YadA↓, Ail↓, OmpX↓, OmpC↓, FlhDC | 4.16 x 10^6^ | 1.98 x 10^6^ | 2.68 x 10^4^ | 2.02 x 10^2^ | 0.005±0.003 | Sensitive |
| AR4 | YadA, Ail↑, OmpX↓, OmpC (-), FlhDC↓ | 1.91 x 10^6^ | 3.07 x 10^6^ | 3.15 x 10^6^ | 3.55 x 10^6^ | 186±32 | Resistant |
| AR4 (25^o^C) | YadA, Ail↑, OmpX↓, OmpC (-), FlhDC↓ | 4.11 x 10^6^ | 1.53 x 10^6^ | 1.00 | 1.00 | 0.000025±0.000004 | Sensitive |
| AR8 | Ye9 wt | 1.30 x 10^6^ | 1.10 x 10^6^ | 5.53 x 10^5^ | 3.05 x 10^5^ | 23±10 | Intermediate |
| Ye9c | Ye9 YadA (-) | 3.48 x 10^6^ | 3.54 x 10^6^ | 5.68 x 10^5^ | 1.28 x 10^3^ | 0.04±0.03 | Sensitive |
| Ye12 | Ye9 Ail (-) | 7.09 x 10^5^ | 9.31 x 10^4^ | 1.92 x 10^4^ | 1.78 x 10^3^ | 0.3±0.1 | Sensitive |
| Ye13 | Ye9 OmpX (-) | 2.47 x 10^6^ | 8.26 x 10^5^ | 5.68 x 10^5^ | 9.61 x 10^4^ | 4±0 | Intermediate |
| OP3 | Ye9 OmpC (-) | 1.73 x 10^6^ | 2.37 x 10^5^ | 9.60 x 10^4^ | 1.09 x 10^4^ | 0.6±0.3 | Sensitive |
| OP3/pBBRC4 | Ye9 wt | 2.00 x 10^6^ | 1.41 x 10^6^ | 1.62 x 10^6^ | 3.38 x 10^5^ | 17±0 | Intermediate |
| DN1 | Ye9 FlhDC (-) | 3.11 x 10^6^ | 3.52 x 10^6^ | 3.22 x 10^6^ | 2.41 x 10^6^ | 77±27 | Resistant |
| DN1 (25^o^C) | Ye9 FlhDC (-) | 2.0 x 10^6^ | 1.7 x 10^6^ | 1.5 x 10^6^ | 1.5 x 10^4^ | 1.0135±0.28 | Sensitive |
| DN1/pBF | Ye9 wt | 3.75 x 10^6^ | 4.01 x 10^6^ | 2.15 x 10^6^ | 5.43 x 10^5^ | 15±6 | Intermediate |
| AR4c | AR4YadA (-) | 1.63 x 10^6^ | 7.91 x 10^5^ | 1.33 x 10^5^ | 2.25 x 10^1^ | 0.001±0.001 | Sensitive |
| AR7 | AR4 Ail (-) | 7.51 x 10^5^ | 5.62 x 10^5^ | 3.35 x 10^5^ | 5.25 x 10^4^ | 7±3 | Intermediate |
| AR4/pBF | AR4 FlhDC↑ | 2.08 x 10^6^ | 2.07 x 10^6^ | 2.14 x 10^6^ | 8.39 x 10^5^ | 40±7 | Intermediate |
| AR4/pBF (25^o^C) | AR4 FlhDC↑ | 1.25 x 10^6^ | 1.17 x 10^6^ | 1.24 x10^5^ | 4.05 x 10^3^ | 0.5±0.24 | Sensitive |

^a^ Level of protein in strains compared with the level in the parent (Ye9 wt or AR4): (-) – not expressed,

↓ – decreased or ↑ – increased

^b^ Colony-forming unit (CFU) – the mean of three determinations

^c^ Contact time of cells with the NHS (T_min_)

^d^ Bacterial survival expressed as a percentage of the T_0_ control value

^e^ Sensitive – ≤1% viable bacteria after 60 min; intermediate/moderate resistance – 1 to 50% viable bacteria after 60 min; resistant – >50% viable bacteria after 60 min
