# Supplementary material for: Regulatory Protein OmpR Influences the Serum Resistance of Yersinia enterocolitica O:9 by Modifying the Structure of the Outer Membrane
Source: PLoS One. 2013 Nov 19;8(11):e79525. doi: 10.1371/journal.pone.0079525 (PMC3834241; doi:10.1371/journal.pone.0079525)
Supplement: Table S2 — Survival of bacterial cells in heat-inactivated serum (HIS). (DOC) [file pone.0079525.s002.doc]

**Table S2. Survival of bacterial cells grown at 37^o^C (unless indicated as 25^o^C) in heat-inactivated serum (HIS)**

| **Strain** | **CFU^a^/ml** | | | |
| --- | --- | --- | --- | --- |
|  | **T_0_^c^** | **T_15_** | **T_30_** | **T_60_** |
| Ye9 wt | 1.81 x 10^6^ | 3.04 x 10^6^ | 4.12 x 10^6^ | 4.94 x 10^6^ |
| Ye9 wt (25^o^C) | 3.30 x 10^6^ | 3.44 x 10^6^ | 3.93 x 10^6^ | 5.26 x 10^6^ |
| AR4 | 2.46 x 10^6^ | 2.64 x 10^6^ | 4.89 x 10^6^ | 6.29 x 10^6^ |
| AR4 (25^o^C) | 2.95 x 10^6^ | 3.25 x 10^6^ | 3.60 x 10^6^ | 5.47 x 10^6^ |
| AR8 | 1.15 x 10^6^ | 1.21 x 10^6^ | 1.84 x 10^6^ | 2.83 x 10^6^ |
| Ye9c | 2.05 x 10^6^ | 4.15 x 10^6^ | 5.62 x 10^6^ | 7.90 x 10^6^ |
| AR4c | 1.37 x 10^6^ | 2.00 x 10^6^ | 3.31 x 10^6^ | 3.41 x 10^6^ |
| Ye12 | 4.93 x 10^6^ | 4.51 x 10^6^ | 4.71 x 10^6^ | 5.41 x 10^6^ |
| Ye13 | 1.43 x 10^6^ | 2.18 x 10^6^ | 2.15 x 10^6^ | 2.08 x 10^6^ |
| OP3 | 1.20 x 10^6^ | 1.53 x 10^6^ | 1.56 x 10^6^ | 1.83 x 10^6^ |
| OP3/pBBRC4^b^ | 1.69 x 10^6^ | 1.81 x 10^6^ | 1.79 x 10^6^ | 2.01 x 10^6^ |
| DN1 | 3.19 x 10^6^ | 3.85 x 10^6^ | 3.73 x 10^6^ | 6.14 x 10^6^ |
| DN1/pBF | 3.78 x 10^6^ | 4.11 x 10^6^ | 5.54 x 10^6^ | 6.31 x 10^6^ |
| AR7 | 5.39 x 10^5^ | 6.41 x 10^5^ | 7.82 x 10^5^ | 1.46 x 10^6^ |
| AR4/pBF | 1.47 x 10^6^ | 1.61 x 10^6^ | 2.16 x 10^6^ | 2.47 x 10^6^ |

No bactericidal activity was observed with any of the examined bacterial strains incubated with HIS (ANOVA, P= 0.12)

^a^ Colony-forming unit (CFU) – the mean of three determinations

^b^ Only one experiment was performed with this strain

^c^ Contact time of cells with the NHS (T_min_)
