# Supplementary material for: Regulatory Protein OmpR Influences the Serum Resistance of Yersinia enterocolitica O:9 by Modifying the Structure of the Outer Membrane
Source: PLoS One. 2013 Nov 19;8(11):e79525. doi: 10.1371/journal.pone.0079525 (PMC3834241; doi:10.1371/journal.pone.0079525)
Supplement: Table S3 — Oligonucleotide primers used for PCR and DNA sequencing in this study. (DOC) [file pone.0079525.s003.doc]

**Table S3. Oligonucleotide primers used for PCR and DNA sequencing in this study.**

| **Primers** | **Primer direction and sequence^a^** |
| --- | --- |
| aA1 | F: 5’-GCTTATGGCACGAAAACGTA-3’ |
| aA976 | R: 5’-CCCTCCATTTAGGTGGGTTT-3’ |
| aA2X | F: 5’-TG**TCTAGA**GCTTATGGCACGAAAACGTA-3’ |
| aA706S | R: 5’-TG**CCCGGG**AGATGGCCCCATTGTTACTG-3’ |
| oX1 | F: 5’-CTGGAATGGCTTTTGGTGAT-3’ |
| oX883 | R: 5’-GATGCAGCGAATGCAGTTAG-3’ |
| oX2X | F: 5’-TG**TCTAGA**AATTTGCGTCTTTCGAGGTG-3’ |
| oX400S | R: 5’-TG**CCCGGG**TTGTCGCTAGCAGATGTTGG-3’ |
| yH1 | F: 5’-TGATGTCATCCCAGGCAATA-3’ |
| yH517 | R: 5’-TGCTGCACCAATCGAGATAC-3’ |
| FaA22 | F: 5’-TTGGGGGAAGGAAGCAAATA-3’ |
| FoX175 | F: 5’-TGGTGATGGATGTCACAAAAA-3’ |
| lacZ3 | R: 5’-GCGAATACCTGTTCCGTCAT-3’ |
| fDC1P | F: 5’-TG**CTGCAG**CCAGGTCTTGGGTGAAAAAT-3’ |
| fDC1072X | R: 5’-TG**TCTAGA**ACTAGCGGGTGACAGAAACG-3’ |

^a^ Direction Forward or Reverse; restriction sites are shown in bold
